# Multivariate Poisson lognormal distribution for modeling counts from modern biological data: An overview

**DOI:** 10.1016/j.csbj.2025.03.017

**Published:** 2025-03-20

**Authors:** Sanjeena Subedi, Utkarsh J. Dang

**Affiliations:** a School of Mathematics & Statistics, Carleton University, Ontario, Canada; b Department of Health Sciences, Carleton University, Ontario, Canada

**Keywords:** Poisson lognormal distribution, Count data, Variational inference, Mixture models, Model-based clustering and classification

## Abstract

Modern biological data are often multivariate discrete counts, and there has been a dearth of statistical distributions to directly model such counts in an efficient manner. While mixed Poisson distributions, e.g., negative binomial distribution, are often the distribution of choice for univariate data, multivariate statistical distributions and their algorithmic implementations tend to have different drawbacks, e.g., non-tractable distributions, non-closed form solutions for parameter estimates, constrained correlation structures, and slow convergence during iterative parameter estimation. Herein, we provide an overview of the Poisson lognormal and multivariate Poisson lognormal distributions. These distributions can be written in an hierarchical fashion. An efficient variational approximation-based parameter estimation strategy as well as a hybrid approach for full Bayesian posterior estimation is available for such models, allowing for scaling up and modeling high-dimensional data. We provide comparisons of the univariate Poisson, the negative binomial, and the Poisson lognormal distributions in terms of the estimated mean-variance relationships using simulations and example real datasets. We also discuss the properties of the multivariate Poisson lognormal distribution, and ability to directly model count data including zero counts, over-dispersion, both positive and negative covariance elements, and the mapping from correlations in the latent space vs. the observed space. Finally, we illustrate their use through two model-based clustering examples using a mixtures of distributions approach in RNA-seq and microbiome data.

## Graphical abstract

**Figure:**
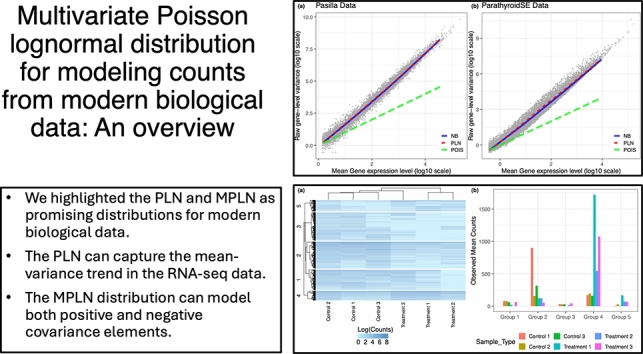

## 1 Introduction

Count data arise in many fields, e.g., omics data, ecology, sports analytics, text mining, etc. These data can be analyzed directly as counts, which preserves the link of interpretation to the data generation mechanism via the use of discrete distributions [44], [32]. However, the practice of using transformed counts with well-characterized distributions and models also continues [26]. This latter approach is reasonable in certain idealized cases, where transformation does not result in loss of much information; this typically happens when the data has no over-dispersion, no zero counts, and counts are not too small [40], [55].

The most ubiquitous distribution for counts is the Poisson distribution but it suffers from the constraint that the mean and variance are equal. However, over-dispersion is often a hallmark of modern biological data where the observed variance is larger than the mean. The most commonly used distribution that can account for over-dispersion is the negative binomial [NB; 21] aka the Gamma-Poisson model. The latter is a mixed Poisson distribution; these types of distributions are an important alternative and include also the Generalized Poisson distribution [13], the Poisson lognormal distribution [PLN; 6], the Poisson inverse-Gamma distribution [56], and Poisson inverse-Gaussian distributions [24], among others. These distributions can be written in a hierarchical parameterization by modeling the mean parameter of the Poisson distribution as a random variable.

Our motivation here is due to the rise of count data from next-generation sequencing technologies in modern biological datasets, e.g., RNA-seq data. These include data on a set of transcripts with high counts, zero counts, over-dispersion, a mean-variance relationship, data normalization (via consideration of library size), and correlation between transcripts. Modeling such data appropriately requires multivariate distributions, which can add further complexity and requirements including numerical and computational constraints, e.g., no closed form parameter estimates. Indeed, previously, independence has been assumed for simplicity when modeling such data [43], which is not ideal. Other challenges that can arise with multivariate implementations are the imposition of particular covariance structures, e.g., some multivariate Poisson distributions only allow for positive correlation between the variables [27].

Reviews of Poisson and discrete distributions are in Karlis and Xekalaki [28] and Nikoloulopoulos and Karlis [39], which note that many univariate mixed Poisson distributions can yield equivalent model fits on data with small counts. The differences between different distributional fits become more important when studying data with larger counts. Nikoloulopoulos and Karlis [39] also notes that mixed Poisson distributions are not frequently used due to numerical and computational challenges. Hence, we will focus our attention on data with larger counts as seen in modern biological datasets.

The multivariate Poission lognormal [MPLN; 2] distribution is a promising distribution, allowing for an arbitrary signed correlation matrix. Recently, computational challenges for this distribution have largely been solved using variational algorithms and the MPLN distribution has been started to be used in different frameworks: network inference [9], probabilistic PCA [8], discriminant analysis [10], time series [12], time series segmentation [14], longitudinal clustering [50], matrix variate data clustering aka three-way clustering [48], cluster analysis in low [49], [51] and high dimensions [42].

Here, we provide an introduction to the univariate Poisson lognormal distribution [5], [6], discuss its relationship and provide comparisons to the Poisson and NB distributions, and compare them via modeling of simulated and real datasets. We then provide an overview of the MPLN distribution as suited for modeling of count data without transformation, modeling over-dispersion and correlation between variables.

The manuscript is structured as follows. Section 2 introduces the PLN distribution, and provides a comparison to the Poisson and negative binomial distributions via some simulations and example datasets. Section 3 provides an overview of the MPLN distribution, discusses the relationship between correlation in the observed vs. latent space, and discusses parameter estimation strategies. Section 4 provides some cluster analysis results, and the manuscript concludes with some ideas for future work and a discussion.

## 2 Univariate Poisson lognormal distribution

Suppose we have *n* observations and $Y_{i}$ is the observed count of the $i^{th}$ observation with i=1,…,n. A univariate PLN [5], [6] distribution is a hierarchical Poisson distribution such that

$$Y_{i}|X_{i}∼\mathrm{Poisson}(e^{X_{i}})\;\text{and}\;X_{i}∼N(\mu ,\sigma^{2}),$$

$\mathrm{Poisson}(e^{X_{i}})$ denotes a Poisson distribution with mean $e^{X_{i}}$, $N(\mu ,\sigma^{2})$ denotes a univariate distribution with mean *μ* and variance $\sigma^{2}$. Due to this hierarchical structure, the mean and variance of the *Y* [5] are

$$E(Y)=e^{\mu +\frac{1}{2}\sigma^{2}}\;\text{and}\;V(Y)=E(Y)+E\;{\left(Y_{i}\right)}^{2}(e^{\sigma^{2}}-1).$$

As opposed to a traditional Poisson model, which imposes a mean-variance relationship such that E(Y)=V(Y), in a PLN model, V(Y)>E(Y). Thus, a PLN distribution can model the over-dispersion encountered in newer biological datasets. The term $\left(e^{\sigma^{2}}-1\right)$ controls the over-dispersion: when $\sigma^{2}=0$ (i.e., X=μ with probability 1), the PLN distribution converges to a Poisson distribution.

The negative binomial distribution is the most widely used distribution for modelling RNA-seq datasets due to its (i) convenient interpretability of the mean and dispersion parameters, (ii) ability to capture the mean-variance relationship observed in the RNA-seq datasets, and (iii) closed-form expression of the probability mass function which facilitates efficient parameter estimation. The negative binomial distribution arises as the marginal distribution of the hierarchical Poisson-Gamma distribution [21], and assumes that

$$Y_{i}|X_{i}∼\mathrm{Poisson}(X_{i})\;\text{and}\;X_{i}∼\mathrm{Gamma}(\alpha ,\beta ).$$

Similar to the PLN distribution, the mean and variance of the *Y* arising from this negative binomial distribution are

$$E(Y)=\alpha \beta \;\text{and}\;V(Y)=E(Y)+E\;{\left(Y\right)}^{2}\left(\frac{1}{\alpha }\right).$$

Thus, in the negative binomial case, $\frac{1}{\alpha }$ controls the over-dispersion. When *α* becomes large, the NB distribution approaches a Poisson distribution. As seen above, the NB and PLN distributions share a similar convenient interpretability of the mean and dispersion parameters. In Section 2.1, we provide a comparison of the fit of the PLN distribution with the NB and Poisson distributions through simulation studies and we demonstrate using two RNAseq datasets that the PLN distribution can capture the mean-variance relationship of RNA-seq data in Section 2.2. Although PLN and its multivariate extension MPLN do not have closed-form expressions for the probability mass functions, recently, an efficient framework for parameter estimation has been proposed using variational approximations, which is outlined in Section 3.2.

### 2.1 Comparison of PLN with Poisson and NB distribution via simulations

Simulation studies were conducted on a series of datasets generated from the univariate Poisson, univariate negative binomial (with varying levels of dispersion), and univariate Poisson lognormal distributions (with varying levels of dispersion resulting in different mean/variance ratios) to compare how different models perform in capturing the mean and variances of these data sets. We generated 100 data sets of size N=1000 from each of the scenarios provided in Table 1. All three distributions were fitted to all 100 datasets generated under each of the nine simulation settings. The estimated mean and variances were obtained from the three models. The average of the estimates of the means and variances along with the standard errors of the estimates from the 100 datasets under each scenario are provided in Appendix Table 2.

**Table 1:** Simulations with 100 datasets each. The generating distribution is provided along with the means and variances used to generate the datasets.

Scenario	Distribution	Mean	Variance
Simulation 1	Poisson	1000	1000

Simulation 2	Negative binomial	1000	5000
Simulation 3	Negative binomial	1000	10000
Simulation 4	Negative binomial	1000	20000
Simulation 5	Negative binomial	1000	50000

Simulation 6	Poisson lognormal	1000	5000
Simulation 7	Poisson lognormal	1000	10000
Simulation 8	Poisson lognormal	1000	20000
Simulation 9	Poisson lognormal	1000	50000

**Fig. 1:**
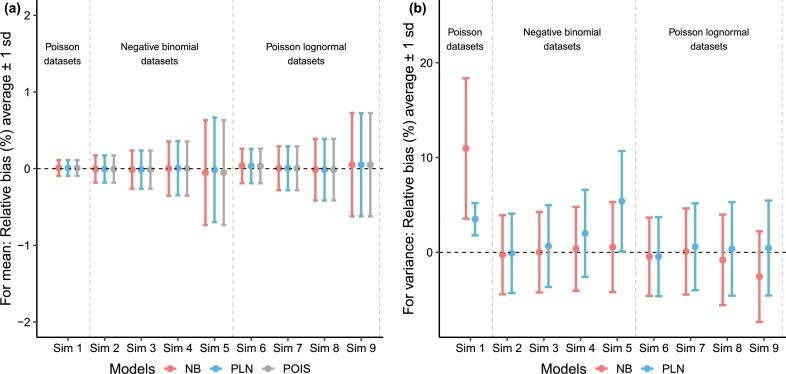
Average of the relative bias (in percentage) ± 1 standard deviations (sds) for the estimation of mean and variance computed using 100 datasets from all nine simulation studies. Note that we omit Poisson distribution for visualization for variance as the Poisson distribution assumes that the mean and variance are the same, and so error for the Poisson distribution was extremely high when the data was over-dispersed. Abbreviations: NB: negative binomial; POIS: Poisson; PLN: Poisson lognormal.

As shown in Fig. 1, all three models captured the mean very well in all nine simulation settings. The relative bias for *θ* (in percentage; defined as $\frac{\overset{ˆ}{\theta }-\theta }{\theta }\times 100\text{\%}$) where $\overset{ˆ}{\theta }$ is an estimate is computed for all 100 datasets from all nine simulation scenarios. The average value of the relative bias (in percentage) is very close to 0 and the standard deviation of the relative bias is less than 1% in all simulation settings. On the other hand, when the data were generated from a Poisson distribution (i.e., Simulation Setting 1), both the PLN and NB models overestimated the variance, with higher average relative bias (in percentage) for the NB model than for the PLN model. When the data were generated from a negative binomial distribution, the PLN model overestimated the variance as dispersion increased. On the other hand, when the data were generated from a Poisson lognormal distribution, the NB model underestimated the variances.

**Fig. 2:**
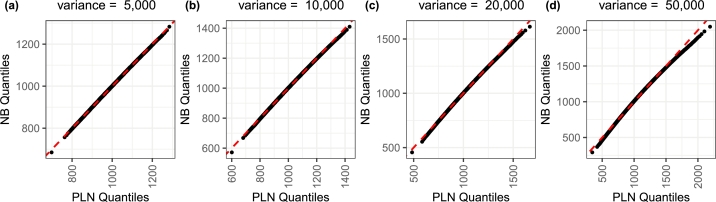
Empirical qq-plot of the PLN distribution against the NB distribution (data generating distribution here) for varying levels of over-dispersion (mean=1000).

We also compared the empirical distributions of the samples generated from the negative binomial distribution against the samples generated from the Poisson lognormal distribution with the same sets of means and variances as Table 1. The distributions look very similar when the over-dispersion is lower but as the variance increases, the distributions seem to show more divergence in the tails (Fig. 2).

### 2.2 Comparison of PLN with Poisson and NB distribution via real data

In most modern biological datasets, the variance tends to increase as the mean increases. A Poisson model, which assumes that the mean and variances are equal, will fail to capture this mean-variance relationship. Thus, the negative binomial distribution has emerged as a widely used univariate distribution of choice. Here, we demonstrate that the PLN distribution can also effectively capture the mean-variance trend observed in RNAseq datasets. We compare the fit of the negative binomial distribution with that of the PLN distribution in capturing the mean-variance relationships on two example RNAseq datasets:

- Pasilla data [4] from the R package pasilla [25].
- ParathyroidSE data from the R package parathyroidSE [22].

**Fig. 3:**
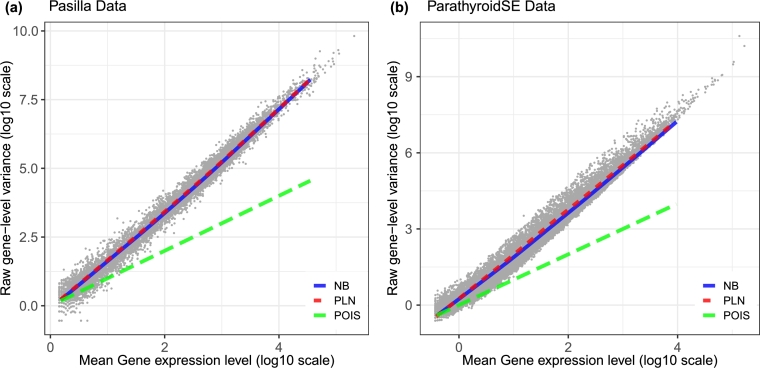
Comparison of the estimated mean-variance relationships on two RNAseq datasets for the Poisson, negative binomial, and Poisson lognormal distributions.

As evident from Fig. 3, the PLN and NB distributions capture the mean-variance relationship very well in both datasets, however, the Poisson distribution fails to capture the mean-variance relationship.

## 3 Multivariate Poisson lognormal distribution

Suppose we have *n* observations and $Y_{i}=(Y_{i1},\ldots ,Y_{id})$ is a *d*-dimensional vector of observed counts of the $i^{th}$ observation with i=1,…,n and $X_{i}=(X_{i1},\ldots ,X_{id})$ is a *d*-dimensional vector of latent variable corresponding to the $i^{th}$ observation, a multivariate Poisson lognormal distribution [2] is a hierarchical Poisson model such that

$$Y_{ij}|X_{ij}∼\text{Poisson}(e^{X_{ij}})\;\text{and}\;X_{i}∼N(\mu ,\Sigma ),$$

where N(μ,Σ) denotes a *d*-dimensional multivariate distribution with mean ***μ*** and variance **Σ** and j=1,…,d. Due to this hierarchical structure, the mean and variance of the **Y** [2], [19] are

$$E(Y_{j})=e^{\mu_{j}+\frac{1}{2}\Sigma_{jj}}\;\text{and}\;V(Y_{j})=E(Y_{j})+E\;{\left(Y_{j}\right)}^{2}(e^{\Sigma_{jj}}-1),$$

where $Y_{j}$ and $\mu_{j}$ are the $j^{th}$ entry of **Y** and ***μ***, respectively, and $\Sigma_{jj}$ is the $j^{th}$ diagonal entry of the matrix **Σ**. An attractive property of the MPLN distribution is that the covariance and the correlation terms also have a closed form:

$$\text{Cov}(Y_{j},Y_{k})=E(Y_{j})E(Y_{k})(e^{\Sigma_{jk}}-1),\text{Cor}(Y_{j},Y_{k})=\frac{e^{\Sigma_{jk}}-1}{{\left[\left\{e^{\Sigma_{jj}}-1+E\;{\left(Y_{j}\right)}^{-1}\right\}\left\{e^{\Sigma_{kk}}-1+E\;{\left(Y_{k}\right)}^{-1}\right\}\right]}^{\frac{1}{2}}},$$

where $\Sigma_{jj}$ is the $j^{th}$ diagonal entry of the matrix **Σ** and $\Sigma_{jk}$ is the entry in the $j^{th}$ row and $k^{th}$ column of the matrix **Σ** such that j≠k.

### 3.1 Importance of covariance structure

**Fig. 4:**
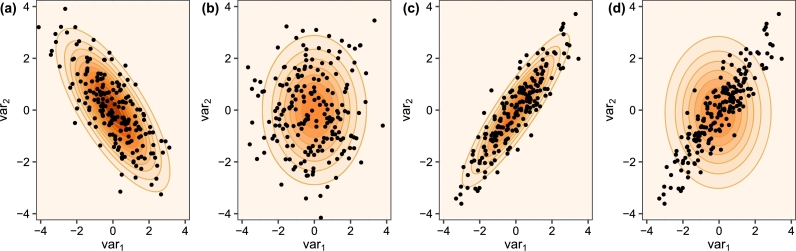
Density contour plots of bivariate data showing (a) negative correlation, (b) no correlation, (c) positive correlation, and (d) fitting a misspecified covariance matrix on positive correlated data points.

Independent negative binomial distributions have previously been utilized to model multivariate data [47], [33], often due to the computational cost of fitting multivariate extensions of these models [45]. However, misspecifying the covariance structure can have a detrimental effect on the fit of the model. We provide examples using two-dimensional toy data sets in which the data are generated from a Gaussian distribution with the same bivariate mean ($\mu_{1}=0$, $\mu_{2}=0$) and variances ($\sigma_{1}^{2}$=2, $\sigma_{2}^{2}$=2) but different correlation structures (with correlations -0.75, 0, and 0.9 in Figs. 4 (a), (b), and (c), respectively).

The contours in Figs. 4 (a), (b), and (c) show the fit of the data using the generating model. While the mean and variances are the same, the spread of the data in the three cases is very different. In Fig. 4 (d), we fit a Gaussian distribution with a spherical covariance structure (i.e., a model that assumes no correlation between variables) to the dataset generated with a positive correlation between the two variables. The true means ($\mu_{1}=0$, $\mu_{2}=0$) and variances ($\sigma_{1}^{2}$=2, $\sigma_{2}^{2}$=2) used to generate the dataset in Fig. 4 (d) and the estimated means (${\overset{ˆ}{\mu }}_{1}=-0.05$, ${\overset{ˆ}{\mu }}_{2}=-0.09$) and variances (${\overset{ˆ}{\sigma }}_{1}^{2}$=2.15, ${\overset{ˆ}{\sigma }}_{2}^{2}$=2.15) were fairly close. However, while the fitted model was able to capture the mean and variance fairly well, the contours of the fitted model in Fig. 4 (d) show that the model does not provide a good fit to the dataset. Thus, the correct specification of a covariance structure is important.

An attractive property of the MPLN distribution is that there is a one-to-one correspondence between the sign and magnitude of the covariance of the latent Gaussian space with that of the observed space, and its ability to model both positive and negative correlations. Furthermore, when the variables are uncorrelated in the observed space, the variables in the latent space will also be uncorrelated, and vice versa, i.e., when $\Sigma_{jk}=0$, $\text{Cov}(Y_{j},Y_{k})$ will also be 0. [2] notes that for the MPLN model:

$$|\text{Cov}(Y_{j},Y_{k})|<\;|\text{Cov}(X_{j},X_{k}))|.$$

Thus, the range of possible correlations in the observed space will not be as wide as that of the latent space. However, when the means of the two variables are large, the correlations of the observed and latent space can be quite close [2]. To demonstrate this, we conducted a set of simulations to empirically show the range of correlations of observed and latent space for a range of values for mean and variances. Note that the correlation in the latent space is

$$\text{Cor}(X_{j},X_{k})=\frac{\text{Cov}(X_{j},X_{k})}{{\left[\text{Var}(X_{j})\text{Var}(X_{k})\right]}^{\frac{1}{2}}}=\frac{\Sigma_{jk}}{\sqrt{\Sigma_{jj}\Sigma_{kk}}},\;\text{thus}\;\Sigma_{jk}=\text{Cor}(X_{j},X_{k})\sqrt{\Sigma_{jj}\Sigma_{kk}}\;.$$

Hence, we can write the correlation in the observed space as

$$\text{Cor}(Y_{j},Y_{k})=\frac{e^{\Sigma_{jk}}-1}{{\left[\left\{e^{\Sigma_{jj}}-1+E\;{\left(Y_{j}\right)}^{-1}\right\}\left\{e^{\Sigma_{kk}}-1+E\;{\left(Y_{k}\right)}^{-1}\right\}\right]}^{\frac{1}{2}}},=\frac{e^{\text{Cor}(X_{j},X_{k})\sqrt{\Sigma_{jj}\Sigma_{kk}}}-1}{{\left[\left\{e^{\Sigma_{jj}}-1+E\;{\left(Y_{j}\right)}^{-1}\right\}\left\{e^{\Sigma_{kk}}-1+E\;{\left(Y_{k}\right)}^{-1}\right\}\right]}^{\frac{1}{2}}}.$$

**Fig. 5:**
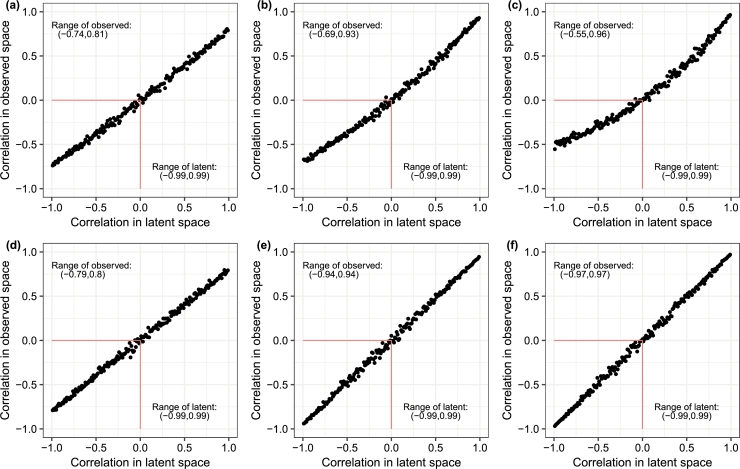
Each figure provides the range of Spearman correlation in the latent space vs the range of Spearman correlation in observed space from a bivariate PLN distribution. There is a one-to-one mapping of zero correlation. In (a), (b), and (c), the true mean was low value of 50 and variance/mean ratio were set to 5, 20, and 50, respectively while in (d), (e), and (f), the true mean was higher value of 10,000 and variance/mean ratio were again set to 5, 20, and 50, respectively.

As the relationship between the correlation of the observed variables and the latent variables is monotonic but not linear, we utilize Spearman correlation instead of Pearson correlation. In simulation settings in Figs. 5 (a), (b), and (c), the true means were set to a low value of 50 and the variance/mean ratios were set to 5, 20, and 50, respectively. In simulation settings in Figs. 5 (d), (e), and (f), the true means were set to a high value of 10,000 and the variance/mean ratios were again set to 5, 20, and 50, respectively.

As evident from Fig. 5, when the mean is large, the Spearman correlation in the observed space can span almost the same range as the observed Spearman correlation in the latent space. Aitchison and Ho [2] also note that the marginal distribution of an MPLN is a PLN and that the marginal distributions are independent PLN distributions if the covariance parameter is zero. Therefore, the multivariate Poisson lognormal distribution is a promising distribution due to its ability to model a wide range of correlation structures.

We further demonstrate the ability of the MPLN distribution to capture the true correlations when modelling uncorrelated multivariate discrete datasets and correlated multivariate discrete datasets through simulation studies.

Modelling uncorrelated multivariate discrete data using MPLN: We conducted a set of simulations to demonstrate the performance of the MPLN distribution in modelling uncorrelated discrete data from three different scenarios: (A) multivariate datasets generated using independent Poisson distributions, (B) multivariate datasets generated using independent negative binomial distributions, and (C) multivariate datasets generated using independent univariate PLN distributions.

Modelling correlated multivariate discrete data using MPLN: We conducted another set of simulations to investigate the performance of MPLN distribution in modelling correlated discrete data from three different scenarios: (D) datasets with all positive correlations in the observed space, (E) datasets with all negative correlations in the observed space, and (F) a mixture of both. In all three settings here, the datasets were generated using an MPLN distribution.

**Fig. 6:**
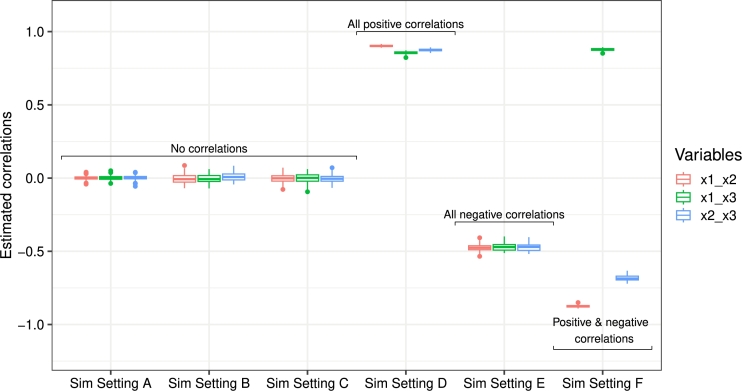
Estimated correlations of the observed variables **Y** from the six simulation settings: Sim settings A, B, and C with uncorrelated variables, and Sim settings D, E, and F with all positive, all negative, and both positive and negative correlations between the variables, respectively.

For each scenario, we generated 100 datasets and fitted an MPLN distribution. The average of estimated means and variances along with their standard errors is provided in Appendix Table 3, which shows that the MPLN can capture the mean and variances of the uncorrelated multivariate datasets generated from independent Poisson distributions, independent NB distributions, and independent PLN distributions. Visualization of the estimated correlation terms for all six simulation settings is provided in Fig. 6. In the datasets where the variables were independent of each other, the estimated correlations from the MPLN distribution were close to 0 in all three scenarios. Furthermore, in the scenarios where the variables were positively and/or negatively correlated, the MPLN distribution was still able to recover the underlying correlation structures.

### 3.2 Recent developments in parameter estimation

A reviewer noted that lack of a closed-form expression for the probability mass function typically implies that expectations may be harder to compute, statistical inference in general may be more complicated, computation of model selection criteria would require approximations or numerical techniques, and there is a risk of errors due to approximations. One of the main limitations to the wider adoption of the MPLN distribution early on has been the computational complexity in fitting these models as the marginal distribution of MPLN is not tractable and the marginal distribution of MPLN involves multiple integrals, which cannot be simplified, resulting in challenges in parameter estimation.

Some recent work has focused on Bayesian approaches for parameter estimation within an expectation-maximization (EM) algorithm [15] utilizing Monte Carlo EM algorithm via importance sampling [19] or Hamiltonian Monte Carlo [49] within the EM-algorithm. However, these approaches were computationally intensive and not ideal for high-dimensional datasets.

More recent studies have utilized variational Gaussian approximations for parameter estimations of MPLN distribution [9], [51]. In variational Gaussian approximation, the complex posterior distribution of the latent variable $X_{i}$ is approximated using a computationally convenient Gaussian distribution obtained by minimizing the Kullback–Leibler (KL) divergence between the true and approximating densities of the latent variable $X_{i}$. The estimates of the parameters and variational parameters are obtained iteratively. Quantities like model selection criteria can then be obtained via the approximation.

Silva et al. [48] compared both the Bayesian and variational approximation-based approaches for parameter estimation for model-based clustering using mixtures of MPLN distributions and showed that variational approximation is substantially faster than the Bayesian approach. While the variational approach brings computational advantages, the expected values of the sufficient statistics involving the unobserved latent variable $X_{i}$ required for parameter estimation are computed using the approximating density of $X_{i}$. On the other hand, in a Bayesian approach, expected values of the sufficient statistics involving the unobserved latent variable $X_{i}$ are computed using the samples from the true posterior distribution of $X_{i}$. To mitigate the limitations of the variational approach, Silva et al. [48] proposed a hybrid approach for parameter estimation in a clustering setting. In the two-step hybrid procedure, the clustering and parameter estimates are obtained using a variational approach in the first step. The cluster membership from the variational approach is treated as the final clustering and the parameter estimation from the variational approach is used as starting values for the Bayesian approach. Then, the model parameters are updated using the full Bayesian approach for the given clustering. As the parameter estimates from variational approach provide a good initial value, the Bayesian approach converges quite quickly. This approach has been shown to be computationally efficient and ensures that the parameter estimates are obtained using the true posterior. Through detailed simulation studies, [48] provide comparisons of the accuracy of the parameter estimations from the three different approaches and demonstrate that the parameters recovered from the hybrid approach are quite comparable to the full Bayesian approach but the computational time needed for the hybrid approach is substantially reduced, thus making the approach scalable for high-dimensional datasets.

## 4 Clustering using MPLN distributions

Here, we highlight the application of the MPLN distribution in cluster analysis. Cluster analysis is widely used in bioinformatics to group observations into homogeneous subgroups or clusters such that observations within a cluster are more similar to each other than to observations in other clusters. Some examples include clustering genes such that genes with similar expression patterns are clustered together [38], [49], [35], clustering microbiome samples such that individuals with similar microbiome compositions are clustered together [52], [36], [17], [53], etc. Recently, a model-based clustering framework using a mixture of MPLN distributions has been developed for clustering RNA-seq data [49], [48], [42]. Model-based clustering assumes that the population is a mixture of subpopulations where each subpopulation can be modelled using a statistical distribution. A *G*-component mixture of MPLN distributions can be written as

$$f(Y)=\sum_{g=1}^{G}\pi_{g}f(Y;\mu_{g},\Sigma_{g}),$$

where $f(Y;\mu_{g},\Sigma_{g})$ is the probability mass function of the $g^{th}$ component with parameters $\mu_{g}$ and $\Sigma_{g}$ to model the $g^{th}$ subpopulation and $\pi_{1},\ldots ,\pi_{G}$ are the mixing weights. Assuming that each cluster corresponds to a component, probabilities of the observations belonging to these *G* components or clusters are computed and observations are assigned to the cluster with the maximum a-posteriori probability. Typically, in clustering, the number of components is unknown. Hence, the models are fitted for a range of different numbers of components and the best-fitting model is selected using a model selection criterion. Herein, we used Bayesian information criteria [BIC; 46] for model selection.

Here, we demonstrate the application of mixture of MPLN distributions for clustering two main sources of multivariate discrete data in bioinformatics: RNAseq data and microbiome data.

### 4.1 Clustering RNAseq data using mixtures of MPLN distributions

In RNAseq studies, the abundance of mRNA transcripts in different samples can be quantified using next-generation sequencing technologies. We applied the mixtures of MPLN [49], [51] to cluster the gene expression values from the Zebrafish dataset originally published in [18] and available from the R package zebrafishRNASeq. The study investigates the mechanisms of gene regulation for odorant receptor gene silencing in olfactory sensory neurons (OSN) in zebrafish. The RNAseq dataset was obtained from three pairs of gallein-treated and controlled embryonic zebrafish pools and consisted of abundances of 32561 genes across these six samples. We focused on the subset of genes identified by [18] as differentially expressed and with an absolute value of the log-fold change greater than 2. The resulting dataset comprised 2069 genes.

**Fig. 7:**
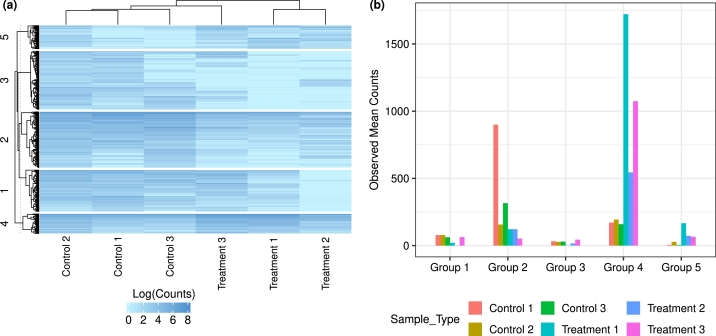
Cluster analysis of the gene expression values from the Zebrafish dataset using a mixtures of MPLN approach. The left subpanel shows the log-transformed abundances of the genes in the 5 inferred clusters in the selected model, while the right subpanel shows the average mean counts of the samples in different clusters.

We utilized a mixture of MPLN distributions with parameter estimation using a variational EM framework by [51] and incorporated a normalization constant for the RNAseq dataset as in [49]. The normalization constant was computed using the trimmed mean of M values (TMM) approach implemented in the R package edgeR [44]. We fitted the mixtures of MPLN distributions with G=1 to 10 and the BIC selected a five-component (G=5) model as the best fitting model. Visualization of the log-transformed abundances of the genes in different clusters is provided in Fig. 7 (a) and the average mean counts of the samples in different clusters are provided in Fig. 7 (b).

Each cluster has a distinctive expression profile. Cluster 2 seemingly has high abundances for control samples whereas Cluster 4 has high abundance for gallein-treated samples. Similarly, Cluster 3 also has relatively high values for control samples and Cluster 5 has relatively high values for the gallein-treated samples, however, their abundances are much lower compared to genes in Clusters 2 and 4. Finally, Cluster 1 on the other hand has a higher abundance for all samples except for Treatment 2.

### 4.2 Clustering microbiome data using mixtures of MPLN distributions

In microbiome studies, the abundance of various microorganisms in a sample can be obtained using next-generation sequencing technologies. Typically, such data are treated as compositional (observed values are strictly positive and constrained by their sum) as they can reveal relative abundance [20]. A natural model for compositional count data is a multinomial model, however, it fails to capture the observed variability in the microbiome data. Thus, Dirichlet-multinomial distribution-based approaches have been widely used to cluster microbiome data [7], [52], [23] However, the limited number of parameters in the DM model may not adequately capture the complex covariance pattern observed in the microbiome data [54]. Furthermore, the Dirichlet multinomial distribution also imposes the restriction that the correlation between the variables (i.e., taxa) is negative. An alternative approach is to use a distribution that utilizes a log-ratio transformation such as additive log-ratio [ALR; 1], centred log-ratio [CLR; 1], or isometric log-ratio [ILR; 16]; this maps the compositions from a restricted simplex to open real space and complex correlations can be modelled in this real space [41]. However, these models suffer from the disadvantages that these log-ratio transformed values are either hard to interpret, or they lose that one-to-one correspondence with the observed space and can be impacted by the choice of the reference variable, or can result in a singular covariance matrix [41].

Here, we demonstrate that with an appropriate normalization, mixtures of MPLN can be used to cluster microbiome data. Advantages that the use of MPLN offers for modeling microbiome data include the ability to handle large discrete count values, modeling both positive and negative correlations, and the latent space having a one-to-one correspondence with the observed space, the latter making inference from latent space to observed space easier. For illustration, we use the dataset atlas1006 [30] from the R package microbiome [31]. The dataset comprises genus-level microbiome profiling of the faecal samples from 1151 healthy (i.e., no known health complications) individuals from 15 Western countries from Europe and the United States. To account for compositional nature of the microbiome data, we include the normalization factors as an offset term. Similar to RNAseq data, it normalizes the samples for differing sequencing depths between the samples. Following the analysis pipeline of widely used R package phyloseq [37] for microbiome data, we compute the normalization factors using the R package DESeq2 [34] and incorporated the normalization factors within the MPLN framework similar to [49]. We selected the top five most abundant genera for cluster analysis.

**Fig. 8:**
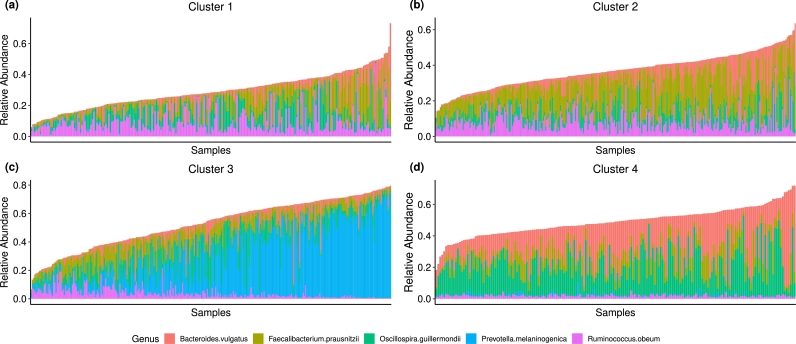
Cluster analysis of the top five most abundant genera from the atlas1006 dataset using a mixtures of MPLN approach. Each subpanel shows different clusters. Stacked barplots of relative abundances for each sample are provided for the five most abundant taxa only (and therefore do not sum to one).

We fitted the MPLN mixture model with G=1 to 5 and used BIC for model selection. A four-component model (corresponding to a model with 4 clusters) was selected as the best-fitting model. Visualization of the relative abundance (computed with respect to the total abundance of the sample) of the five different bacterial taxa in the estimated clusters is provided in Fig. 8.

Each cluster has its own distinctive signature. Cluster 3 comprises individuals with a high relative abundance of *Prevotella melaninogenica* and individuals in cluster 4 have a high abundance of *Oscillospira guillermondii* and *Bacteroides vulgatus*. Cluster 1 and 2 are similar concerning the abundance of *Ruminococcus obeum* (higher relative abundance in both clusters) and *Oscillospira guillermondii* (lower relative abundance in both clusters) but different for the relative abundance of *Faecalibacterium prausnitzii*. Finally, the relative abundance of *Faecalibacterium prausnitzii* is typically higher in individuals in Cluster 4 compared to that of Cluster 2.

## 5 Conclusions

In this manuscript, we provide an overview of the Poisson lognormal (PLN) and multivariate Poisson lognormal (MPLN) distributions, which are promising distributions for count data. The PLN distribution better models over-dispersion, removing the Poisson distribution's mean-variance identity constraint. We also demonstrate the similarity of the univariate negative-binomial distribution and the univariate Poisson lognormal distribution in modeling over-dispersion.

In addition to over-dispersion, the MPLN distribution possesses the desirable mathematical property of a direct correspondence between the correlation in the latent vs observed space, including zero correlations, and it allows for modeling an arbitrary signed correlation structure. This is in comparison to, for example, the mixed bivariate Poisson distribution of the first kind [29] which can only model positive correlation structures [28]. The MPLN distribution possesses closed form mean, covariance, and the correlation matrices. The use of these distributions is facilitated through software implementations for cluster analysis of multi- and matrix-variate data including full Bayesian and faster variational inference implementations in the MPLNClust [49] and for clustering of high-dimensional data in the mixMPLNFA R packages [48].

Principal component analyses, dimension reduction, and supervised classification are available through PLNmodels [11], and pyPLNmodels [3]. We illustrated use of the MPLN distributions via two implementations of model-based cluster analysis, one for RNAseq data, and another for microbiome data using a mixtures of distributions approach. Important future directions include extensions to account for covariates via the latent Gaussian space and to handle sparsity (zero and low counts). In summary, the PLN and MPLN distributions better model the data generation process, directly use the count data rather than rely on transformation, model over-dispersion, as well as, allow for modeling the correlation of multivariate data.

## Funding acknowledgments

This research was supported by the National Sciences and Engineering Research Council of Canada grant (2021-03812 - Subedi; 2022-04889 - Dang), and the 10.13039/501100001804Canada Research Chairs Program (CRC-2020-00303 - Subedi).

## CRediT authorship contribution statement

**Sanjeena Subedi:** Writing – review & editing, Writing – original draft, Visualization, Software, Project administration, Methodology, Investigation, Formal analysis, Data curation, Conceptualization. **Utkarsh J. Dang:** Writing – review & editing, Writing – original draft, Visualization, Project administration, Methodology, Investigation, Data curation.

## Declaration of Competing Interest

The authors have no conflict of interest to declare.

## Figures and Tables

**Table 2:** The average of the estimated means and variances along with the standard errors (SE) of the estimates for Simulation settings 1–9.

	Distribution	Parameter	True	Average of Estimate (± SE)		
				Poisson	NB	PLN
Sim. Setting 1	Poisson	Mean	1000	1000.09	1000.09	1000.09
				(± 1.03)	(± 1.03)	(± 1.03)
		Variance	1000	1000.09	1109.56	1034.99
				(± 1.03)	(± 74.10)	(± 17.06)

Sim. Setting 2	Negative binomial	Mean	1000	999.95	999.95	999.95
				(± 1.77)	(± 1.77)	(± 1.77)
		Variance	5000	999.95	4986.93	4994.92
				(± 1.77)	(± 208.58)	(± 209.44)
Sim. Setting 3	Negative binomial	Mean	1000	999.87	999.87	999.88
				(± 2.49)	(± 2.49)	(± 2.49)
		Variance	10000	999.87	10001.50	10065.20
				(± 2.49)	(± 424.54)	(± 431.05)
Sim. Setting 4	Negative binomial	Mean	1000	1000.01	1000.01	1000.06
				(± 3.54)	(± 3.54)	(± 3.54)
		Variance	20000	1000.01	20073.37	20400.53
				(± 3.54)	(± 883.7)	(± 917.00)
Sim. Setting 5	Negative binomial	Mean	1000	999.48	999.48	999.85
				(± 6.85)	(± 6.85)	(± 6.83)
		Variance	50000	999.4	50275.56	52695.81
				(± 6.85)	(± 2375.25)	(± 2649.87)

Sim. Setting 6	Poisson lognormal	Mean	1000	1000.35	1000.35	1000.34
				(± 2.25)	(± 2.25)	(± 2.25)
		Variance	5000	1000.35	4976.87	4977.32
				(± 2.25)	(± 206.51)	(± 208.54)
Sim. Setting 7	Poisson lognormal	Mean	1000	1000.05	1000.05	1000.04
				(± 2.86)	(± 2.86)	(± 2.86)
		Variance	10000	1000.05	10008.96	10058.65
				(± 2.86)	(± 454.51)	(± 458.82)
Sim. Setting 8	Poisson lognormal	Mean	1000	999.87	999.87	999.87
				(± 4.01)	(± 4.01)	(± 4.02)
		Variance	20000	999.87	19841.26	20071.93
				(± 4.01)	(± 955.54)	(± 986.53)
Sim. Setting 9	Poisson lognormal	Mean	1000	1000.52	1000.52	1000.51
				(± 6.74)	(± 6.74)	(± 6.74)
		Variance	50000	1000.52	48717.58	50225.38
				(± 6.74)	(± 2390.61)	(± 2503.40)

**Table 3:** The average of the estimated means and variances along with the standard errors of the estimates for Simulation settings A–F.

**Sim setting A**	**Poisson distribution**	

Mean	True	(1000, 1500,1500)
	Average of the estimates	(1000.03, 1499.86,1500.04)
	Standard error of the estimates	(0.91, 1.17, 1.31)

Variance	True	(1000, 1500, 1500)
	Average of the estimates	(1037.34, 1550.86, 1557.99)
	Standard error of the estimates	(22.05, 31.41, 38.99)

**Sim setting B**	**Negative binomial distribution**	

Mean	True	(1000, 500, 1000)
	Average of the estimates	(1000.20, 499.93, 1000.21)
	Standard error of the estimates	(1.44, 0.97, 2.40)

Variance	True	(2000, 1000, 5000)
	Average of the estimates	(2005.90, 993.61, 4996.66)
	Standard error of the estimates	(90.53, 45.40, 219.72)

**Sim setting C**	**Poisson lognormal**	

Mean	True	(1000, 5000, 5000)
	Average of the estimates	(1000.31, 5000.06, 5000.47)
	Standard error of the estimates	(2.27, 4.96, 3.45)

Variance	True	(5000, 20000, 15000)
	Average of the estimates	(4962.17, 19995.75, 14974.48)
	Standard error of the estimates	(223.67, 986.53, 625.14)

**Sim setting D**	**MPLN - high positive correlations**	

Mean	True	(10000, 10000, 10000)
	Average of the estimates	(10001.10, 10000.17, 10000.31)
	Standard error of the estimates	(15.55, 14.28, 13.18)

Variance	True	(200000, 200000, 200000)
	Average of the estimates	(200215.10, 201615.70, 200294.1)
	Standard error of the estimates	(10272.29, 9633.58, 9460.05)

**Sim setting E**	**MPLN - negative correlations**	

Mean	True	(5000, 5000, 5000)
	Average of the estimates	(4998.80, 5001.85, 4999.42)
	Standard error of the estimates	(8.86, 10.98, 10.21)

Variance	True	(100000, 100000, 100000)
	Average of the estimates	(99713.57, 100205.62, 99669.42)
	Standard error of the estimates	(4731.36, 5208.41, 4415.93)

**Sim setting F**	**MPLN - positive & negative correlations**	

Mean	True	(10000, 10000, 10000)
	Average of the estimates	(10004.56, 9996.50, 10005.23)
	Standard error of the estimates	(21.97, 21.94, 20.91)

Variance	True	(400000, 400000, 400000)
	Average of the estimates	(403442.5, 400423.4, 403211.7)
	Standard error of the estimates	(18644.03, 18767.83, 20231.13)

## Data Availability

All datasets used in the manuscript are publicly available through different R packages.
